# Copper and Colorectal Cancer

**DOI:** 10.3390/cancers16213691

**Published:** 2024-10-31

**Authors:** Maciej Małyszko, Adam Przybyłkowski

**Affiliations:** Department of Gastroenterology and Internal Medicine, Medical University of Warsaw, Banacha 1a, 02-097 Warsaw, Poland; maciej.malyszko@wum.edu.pl

**Keywords:** copper metabolism, cuproptosis, colorectal cancer, cuproplasia

## Abstract

Minerals constitute only 5% of the typical human diet but are vital for health and functionality. Copper, a trace element, is absorbed by the human gut at 30–40% from diets typical of industrialized countries. The liver produces metallothioneins, which store copper. Copper is crucial for mitochondrial respiration, pigmentation, iron transport, antioxidant defense, hormone production, and extracellular matrix biosynthesis. Copper deficiency, often caused by mutations in the *ATP7A* gene, results in Menkes disease, an X-linked recessive disorder. On the contrary, Wilson disease is characterized by toxic copper accumulation. Cuproptosis, a unique form of cell death regulated by copper, is a subtype of necrosis induced by enhanced mitochondrial metabolism and intracellular copper accumulation. This process can reduce the malignant potential of tumor cells by inhibiting glucose metabolism. Therapeutically, copper and its complexes have shown efficacy in malignancy treatments. The disruption of copper homeostasis and excessive cuproplasia are significant in colorectal cancer development and metastasis. Therefore, manipulating copper status presents a potential therapeutic target for colorectal cancer, using copper chelators to inhibit copper formation or copper ion carriers to promote cuproptosis. This review highlights the role of copper in human physiology and pathology, emphasizing its impact on colorectal cancer and potential therapeutic strategies. Future AI-based approaches are anticipated to accelerate the development of new compounds targeting cuproptosis and copper disruption in colorectal cancer.

## Simple Summary

Copper is a vital trace mineral supporting many body functions by acting as a cofactor for essential enzymes in energy production and antioxidant defense. It is mainly absorbed in the small intestine, and both its deficiency and excess can lead to serious health issues like neurological disorders and organ damage. In colorectal cancer (CRC), disruptions in copper metabolism play a significant role in tumor development and spread. A high copper concentration may facilitate cancer cell proliferation, angiogenesis, and metastasis. A newly discovered form of cell death called “cuproptosis” refers to a mitochondrial pathway of cell death triggered by excessive copper exposure and subsequent proteotoxic stress. Cuproplasia describes copper-dependent cell growth and proliferation, including hyperplasia, metaplasia, and neoplasia. Targeting copper concentration in the body is emerging as a promising strategy for CRC treatment. Therapies that reduce copper concentration using chelators or increase copper-induced cell death using ionophores have shown potential to inhibit tumor growth and enhance the effectiveness of existing treatments.

## 1. Copper—Trace Element

Minerals comprise only 5% of the human diet but are crucial for health. Based on the required amount, they are classified into macrominerals, trace elements, and ultra-trace elements. Trace elements, such as copper, fluoride, and iodine, are needed in small amounts (1 to 100 mg/day). In the U.S, adults typically consume 1 to 1.6 mg of copper daily [1]. The body contains 50 to 120 mg of copper, mostly found in the liver, brain, bones, kidneys, heart, and pancreas [2]. Dietary copper comes mainly from vegetables, grains, and legumes (beans, peas, and lentils), providing about 20% of the intake [3]. These foods make up 60% of the daily copper intake in Western diets. Other copper-rich foods include meat, fish, and poultry [3]. Liver has the highest copper content (20 to 180 mg/kg), compared to white bread (1 to 2.6 mg/kg) and cow’s milk (0.02 to 0.08 mg/kg).

## 2. Copper Metabolism

In industrialized countries, the human gut absorbs about 40% of consumed copper. This absorption mainly occurs in the small intestine after food digestion in the stomach and duodenum [2,3,4]. Only a small fraction of dietary copper is absorbed in the stomach [5]. Gastric acid helps to dissolve copper, making it easier to absorb [2]. Proteins and carbohydrates enhance copper absorption, while organic acids and chelating agents also aid this process [5]. Malabsorption conditions and gastrointestinal diseases can impair copper uptake [5,6]. Copper is absorbed through active transport at low intake levels and passive diffusion at high intake levels [5,6,7].

ATP7A-P-type ATPase transfers copper via a secretory pathway for efflux from enterocytes and other cells [7,8]. Ingested copper is bound loosely to albumin and amino acids and transported via the portal vein to the liver. In the hepatocytes, copper is transported by ATP7B into the trans Golgi network or cytosolic vesicles and is further incorporated into ceruloplasmin or excreted with the bile [7]. The liver is a place for metallothionein production, which serves as a copper storage protein. Nearly half of copper elimination from the body occurs through biliary excretion, while the rest is excreted via other gastrointestinal secretagogue [5]. The latter mechanism regulates copper homeostasis and prevents deficiency or overload.

In humans, copper deficiency may manifest as various clinical symptoms, such as myeloneuropathy, edema, leucopenia, hepatosplenomegaly, skin depigmentation, and osteoporosis [9,10].

Mutation in the *ATP7A* gene causes Menkes disease/syndrome, X-linked recessive genetic disorder, and a severe form of copper deficiency, with very early onset in infancy. Low ceruloplasmin, low serum copper concentration, erythrocyte copper-zinc superoxide concentration, and low 24-h urinary copper excretion are present in laboratory data if tests are performed [3].

On the other hand, copper can be toxic when present in excess, potentially causing cirrhosis, liver failure, psychiatric manifestations, and neurological disorders. Wilson disease, a genetic disorder caused by a mutation in the *ATP7B* gene, leads to excessive copper buildup in the liver and other organs. Studies have reported that patients with Wilson disease exhibit decreased expression of duodenal copper transporter 1 (CTR1) mRNA and protein compared to healthy individuals [11]. Simultaneously, the production of ATP7A mRNA and protein is increased. This variation in copper transporter expression may protect against systemic copper overload due to the dysfunction of *ATP7B* [11].

## 3. Biological Role of Copper

Copper has two oxidation states, i.e., reduced Cu (I) and oxidized Cu (II) [12], as it can accept and donate electrons. Copper can switch between these two oxidation states [13], making it essential for several enzymes containing copper with various biological activities, such as cytochrome c oxidase (electron transport), zinc-copper superoxide dismutase (antioxidant defense), lysyl oxidase (involved in cross-linking in collagen), dopamine monooxygenase (neurotransmitter synthesis), ceruloplasmin (copper transporter and ferroxidase), factor V (coagulation), and tyrosinase (melanin production) [13]; therefore, copper plays a crucial role as a cofactor in numerous vital physiological processes.

### 3.1. Cuproplasia

The term cuproplasia describes cell growth or proliferation in terms of dependence on copper. Cell cycle regulation by copper is mediated by signaling pathways using enzymatic and non-enzymatic copper-regulated activities. The traditional role of copper is to serve as a static cofactor of protein function and affect essential cellular pathways.

### 3.2. Copper and Activity of Enzymes

Copper serves dual roles as both a negative and positive allosteric modulator in regulating various enzyme activities [14]. Acting as a negative allosteric regulator, copper influences Unc51-like kinase 1 (ULK1) and ULK2 signaling pathways, thereby augmenting kinase activity under conditions of amino acid scarcity [15]—consequently, this modulation by copper leads to a reduction in autophagic flux. Conversely, as a positive allosteric regulator, copper associates with mitogen-activated protein kinase 1 (MEK1) and MEK2, facilitating the enhanced phosphorylation of extracellular-signal-regulated kinase 1 (ERK1) and ERK2 [16,17]. This interaction subsequently intensifies RAF-MEK-ERK signaling cascades. Copper is also a positive allosteric regulator of PDK1 and CK2 kinases, which in turn modulate the Pi3K/Akt cascade, and also of the STAT signaling pathways [18,19]. Pharmacologically, the signaling influenced by copper-dependent kinases can be modulated with copper chelators, which act as inhibitors, or through metal ion carriers that function as activators [20]. These activators promote the redistribution of copper within cellular and subcellular compartments [21]. Moreover, akin to ferritin’s role as an acute-phase reactant, ceruloplasmin also exhibits increased serum concentration during conditions such as inflammation, pregnancy, coronary artery disease, diabetes, various cancers, and chronic kidney disease, reflecting its acute-phase reactant status [22].

## 4. Colorectal Cancer

Colorectal cancer (CRC) is a prevalent type of cancer that is increasingly diagnosed worldwide. The risk of developing CRC is affected by both genetic and environmental factors. As reported by the World Health Organization’s GLOBOCAN database, CRC is the third most common cancer in men and the second most common in women [23]. There is variation in incidence and mortality in terms of geographical region, with the highest mortality being in Eastern Europe; the highest incidence being in Europe and Australia; and the lowest incidence being in Asia and Africa. These variations are likely due to differences in diet, environmental conditions, socioeconomic status, genetic predisposition, and lower rates of CRC screening [24].

The treatment of advanced colorectal cancer (COAD) includes surgery, targeted therapy, chemotherapy, and immunotherapy. Adjuvant chemotherapy has been shown to lower the risk of recurrence in about two-thirds of patients with stage III disease [25]. Despite recent advancements with targeted and immunotherapies, the prognosis for COAD patients remains bleak, with only 11–15% of those with stage IV metastatic disease surviving for five years [26,27,28]. Current effective treatments include colectomy, radiotherapy, and adjuvant chemotherapy, though their effectiveness is somewhat limited. Patients often suffer from severe side effects, such as neuropathy and diarrhea related to chemotherapy, particularly with oxaliplatin [28,29]. Immunotherapy, thanks to the discovery of anti-PD-1 and PD-L1 antibodies, is a promising new treatment option for CRC patients; however, it has been effective in only a selective number of CRC patients with high-frequency microsatellite instability (MSI-H) or mismatch repair deficiency (dMMR) [30].

For many patients with colorectal cancer (CRC), immunotherapy proves ineffective, likely due to the intricate characteristics of the tumor microenvironment (TME). There is a well-recognized correlation between clinical prognosis, the success of anti-tumor immunotherapy, and the infiltration of tumor-infiltrating lymphocytes (TILs) within tumor tissues [31,32]. Patients with microsatellite instability (MSI) tend to have more TILs in their cancer tissues due to somatic mutations accumulating in their DNA, leading to a more positive response to immunotherapy. Conversely, patients with microsatellite stability (MSS) are more likely to display a diminished response in immunotherapy and TIL infiltration [33,34]. Therefore, the unmet need for research is to search for new immune-related indicators of prognosis and to determine immunotherapy’s role and effectiveness in colorectal cancer. Previously, it has been shown that ferroptosis-related metabolism plays a role in the effectiveness of tumor immunotherapy [35]. Now, it is time to investigate the role of copper metabolism in immunotherapy, as this trace element, due to its redox characteristics, may be both beneficial and harmful to cells [36]. Blockhuys et al. recently reported that tumor tissues require higher copper concentrations for cell proliferation promotion and metastasis than healthy cells [37].

## 5. Cuproptosis and Malignancy

Tsvetkov et al., for the first time, use the term cuproptosis as an unusual model of copper-regulated cell death (RCD). This model is different from apoptosis, ferroptosis, and pyroptosis, as an inflammatory cell death usually caused by microbial infection, accompanied by the activation of inflammasomes and the maturation of pro-inflammatory cytokines interleukin-1β (IL-1β) and interleukin-18 (IL-18), necrosis, and traditional cell death models [38]. Cuproptosis, a necrosis subtype of cell death, results from the increased turnover of mitochondrial-dependent energy [38].

The essential factor of cuproptosis is the direct interaction between copper and the lipidated components of the tricarboxylic acid (TCA) cycle. It enables the cumulation of lipidated proteins and the subsequent deficiency of iron-sulfur cluster proteins. This leads to induction of proteotoxic stress, resulting in cell dysfunction, and finally causing cell death, which is either apoptotic or necrotic [38,39]. In detail, under the regulation of the ferredoxin 1-FDX1 (an iron-sulfur protein that mediates electron transfer in a range of metabolic reactions), LIAS-lipoyl synthase (an Fe-S cluster protein and component of the S-adenosylmethionine (SAM) superfamily that takes part in the final step of lipoic acid biosynthesis) binds lipoyl moiety to dihydrolipoamide S-acetyltransferase-DLAT (a component enzyme of the pyruvate dehydrogenase complex, which is crucial step in linking glycolysis to the TCA cycle) [40,41]. During this glycolysis process, pyruvate metabolizes into acetyl-CoA, which is used in the citric acid cycle to carry out cellular respiration.

An excess of copper in the cell results in copper ions attaching to the lipoyl group of lipoylated DLAT, which leads to DLAT oligomerization, subsequent proteotoxic stress, and, ultimately, to cell death [42]. This phenomenon, known as cuproptosis, is triggered by high levels of intracellular copper, leading to the buildup of mitochondrial lipoylated proteins and the destabilization of Fe–S cluster proteins [43]. Since mitochondria are vital for glycolysis, which is essential for cancer cell growth, cuproptosis can lessen the malignancy of tumor cells by hindering glucose metabolism [36]. The effectiveness of copper and its complexes as cancer treatments has been demonstrated [44,45]. Various mechanisms of cell death have been identified through targeting the Endoplasmic Reticulum with the Copper 2+ Complex causing immunogenic cell death of breast cancer Stem Cells [46], upregulating ULK1 autophagy cell death in colorectal cancer [47]. The tumor microenvironment consists of various immune cells, such as CD4+ T cells, CD8+ T cells, and Treg cells [48], which are essential for developing tumor cells within their surrounding environment, separate from the malignant proliferation of cancer cells. CD4+ T cells support the anti-tumor activity of CD8+ T cells, while Treg cells can suppress it. Treg cells are often significantly associated with prognosis and treatment outcomes. Research by Greten F.R. and Mitra [49,50] has indicated that copper ions are deeply involved in both humoral and cellular immunity. However, the exact role of copper-related genes (CRGs) in influencing immune cells in the tumor microenvironment is not fully understood.

Several recent publications have investigated the relationship between long noncoding RNAs (lncRNAs) and cancer [51,52]. LncRNAs are over 200 bp long and transcribed by RNA polymerase II without the capacity to encode proteins. Nevertheless, they play a vital role in tumor growth and metastasis by affecting gene transcription and post-transcriptional modifications [51,52]. These elements could serve as valuable biomarkers for the early detection, diagnosis, prognosis, and treatment effectiveness in various cancers, including lung, gastric, liver, breast, and colorectal cancers [53,54,55,56,57,58]. The role of lncRNAs involved in copper-induced cell death in cancer progression is still under investigation. In a recent study by Weng et al. [59], a copper cell-death-related lncRNA signature (CRLSig, i.e., AC129926.1, AP002954.1, AC023511.1, LINC01537, and TMEM75) was identified as an innovative prognostic biomarker and potential therapeutic target for patients with gastric cancer. TMEM75, an oncogene, promotes colorectal cancer progression, relying on the activation of SIM2 [60].

Recently, Balihodzic et al. reported a relationship between long noncoding RNA extraction and tumor glucose metabolism [61].

**Figure 1:**
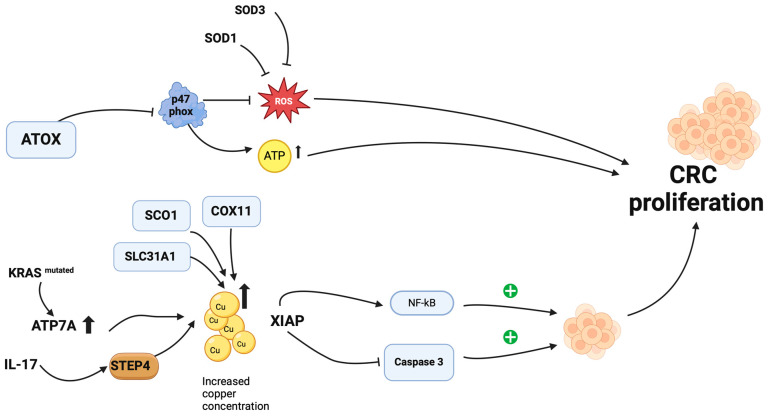
Role of copper in colorectal cancer development. ATOX, Antioxidant-protein 1; SOD1, Super oxide dismutase 1; SOD3, Super oxide dismutase 3; ROS, Reactive oxygen species; SLC31A1, Solute Carrier Family 31 member 1; SCO1, Synthesis of Cytochrome C Oxidase 1; COX11, Cytochrome C Copper Chaperone 11; CRC, colorectal cancer; ATP7A, P-type ATPase; ATP, Adenosine triphosphate; IL-17, Interleukin-17, STEP4, Six-transmembrane epithelial antigen of prostate 4; XIAP, X-linked inhibitor of apoptosis; NFkB, Nuclear factor kappa B. Created in BioRender.com.

Figure 1 depicts how copper homeostasis promotes tumor growth.

## 6. Cuproptosis and Colorectal Cancer

In the development of colorectal cancer, disbalance in copper homeostasis and cuproplasia are pivotal factors [62]. Antioxidant Protein 1 (ATOX1), a human copper metal chaperone, is essential for maintaining copper homeostasis. It is responsible for absorbing cytosolic copper from CTR1 and transporting it to the copper pumps in the trans Golgi network, ultimately delivering it to the ATP7A and ATP7B proteins [63]. Additionally, ATOX1 has been linked to cell proliferation through being a transcription factor and regulating the expression of cyclin D1 [64]. An excessive rise in ATOX1 is associated with CRC proliferation, implying that elevated copper levels result in a concomitant increase in ATOX1. Moreover, activin A, an inflammatory cytokine, causes nuclear localization of ATOX1 in CRC, however, on the other hand, activin A is regulated by ATOX1 [65].

Conversely, the synthesis of reactive oxygen species (ROS) can be suppressed by ATOX, and ATP synthesis may be enhanced as a result of the reduced expression of the NADPH oxidase subunit p47 phox [66]. In this context, cancer cells are capable of development and proliferation. Elevated copper concentration facilitates the influx of copper into colorectal cancer cells, enhancing their proliferation. Specifically, in the context of a KRAS mutation in CRC, excess copper ions in healthy cells are relocated to the cell surface for export via ATP7A. This mechanism allows the KRAS mutant to increase the intracellular copper levels, thereby promoting the expression and stability of *ATP7A* on the cell surface [16,67]. Such an environment renders KRAS mutants dependent on a high copper concentration, further encouraging CRC proliferation.

Additionally, other copper chaperones, such as SCO1 (Synthesis of Cytochrome C Oxidase 1), SLC31A1 (Solute Carrier Family 31 Member 1), and COX11 (Cytochrome C Oxidase Copper Chaperone), may contribute to increased copper influx into the tumor, which is necessary for CRC growth [68].

Furthermore, the relationship between copper status and inflammation also contributes to the proliferation of CRC. The inflammatory cytokine IL-17, which is involved in inflammation and regulates cellular iron and copper homeostasis, is instrumental in promoting STEAP4 (Six-transmembrane epithelial antigen of prostate 4) [69]. IL-17 drives cellular copper uptake via the induction of a metalloreductase, STEAP4. This, in turn, leads to increased intracellular copper and the subsequent activation of E3 ligase XIAP. Then, it potentiates IL-17-induced NFκB activation and suppresses caspase 3 activity. This IL-17-induced STEAP4-dependent cellular copper uptake is critical for tumor formation in an animal model of tumorigenesis in the colon. More importantly, STEAP4 expression correlates with IL-17 level and X-linked inhibitor of apoptosis Protein-XIAP activation in colorectal cancer in humans. Taking these factors together, it creates an environment favorable to the development of colorectal tumors [70]. In summary, inflammation may facilitate colorectal tumor growth through the IL-17-STEAP4-XIAP pathway, with copper playing an essential role in this dynamic [70].

Liu et al. [71] identified a 3-lncRNA signature (LINC00114, LINC00261, and HOTAIR) as a useful candidate for the diagnosis and prognosis of CRC. They also showed that the inhibition of LINC00114 promotes the migratory, invasive, and proliferative abilities of CRC cells.

It has also been demonstrated that the overexpression of LINC00261 relieves cisplatin resistance in CRC cells by promoting apoptosis and inhibiting migration [72].

Similarly, in a recent study, Liu et al. [73] established a prognostic signature incorporating seven cuproptosis-related long-noncoding RNAs (CRLs) and identified three promising diagnostic markers for colorectal cancer (CRC) patients. They downloaded transcriptomic data of 473 colorectal adenocarcinoma tumors, and 41 normal samples were downloaded from the TCGA database (The Cancer Genome Atlas) [74]. Then, they separated the expression of 14,056 lncRNAs and 19,573 mRNAs in colorectal adenocarcinoma samples by Strawberry Perl. Finally, they recruited 417 patients for analysis. They collected 18 cuproptosis-related mRNAs from the previous literature and extracted the expression of those CRGs from cancer samples accordingly. Three lncRNAs—FALEC, AC083967.1, and AC010997.4—were highly expressed in the serum of colorectal adenocarcinoma patients. Based on their findings, they constructed a prognostic signature based on 7 CRLs and found these three promising diagnostic markers for colorectal adenocarcinoma patients. They also found that glucose-related metabolic pathways, closely related to cuproptosis, were enriched in the low-risk group, whereas the immune infiltration scores were lower in the high-risk group. Additionally, they reported that the low-risk group was more sensitive to sorafenib.

This study lays the groundwork for personalized immunotherapy approaches. Separately, Chu et al. [75] created a prognostic model based on cuproptosis-related genes (CRGs) for CRC, which effectively predicted patient prognosis, tumor microenvironment types, and immunotherapy responses. They recognized that AOC3 (Amine oxidase copper-containing 3), CCS (Copper Chaperone for Superoxide dismutase), CDKN2A (Cyclin-Dependent Kinase Inhibitor 2A), COX11 (Cytochrome C Oxidase Copper Chaperone), COX17, COX19, DLD (dihydrolipoamide dehydrogenase), DLAT (dihydrolipoamide acetyltransferase), and PDHB were prognostic CRGs in CRC.

The comprehensive analysis of cuproptosis-related genes (CRGs) revealed that an increased expression of COX17 (Cytochrome C Oxidase Copper Chaperone) in CD4-CXCL13 T cells was observed in CRC, mediating T cell exhaustion and Treg infiltration [75]. DLAT reshapes the TME (tumor microenvironment) by reversing T-cell exhaustion, inducing tumor cell pyroptosis. On the other hand, pyroptosis, induced by oncologic therapy, including chemotherapy, radiotherapy, targeted therapy, and immune therapy, exerts tumor suppression function, evokes anti-tumor immune responses, and potentiates local and systemic anti-tumor immunity. COX17 and DLAT genes were mainly localized in mitochondria, suggesting that cuproptosis was closely related to mitochondrial metabolism. COX17 was expressed in the tumor microenvironment, while DLAT expression levels were low. In addition, COX17 was highly expressed in malignant cells, epithelial cells, fibroblasts, neutrophils, CD4-CXCL13-T cells, CD4-CTLA4-Treg, and CD8-PDCD1-T cells, whereas DLAT was only expressed in cells such as malignant cells, CD4-CCR7-T cells, and CD8-CCR7-T cells. DLAT has the ability to shape the immunoactivity of TME by elevating the cytotoxic T cell levels and reversing T cell exhaustion.

CXCL13, a chemokine from the CXC family, is chemotactic for B cells and, along with its receptor CXCR5, regulates B cell organization within lymphoid tissue follicles, being highly expressed in the lymph nodes, liver, gut, and spleen [76].

In the studies of Chu et al. [75], DLAT expression correlated with CD8^+^ T cells, neutrophils, macrophages, B cells, and dendritic cell infiltration, and DLAT levels were significantly upregulated after anti-PD-1 immunotherapy.

These findings underscore the clinical significance of CRGs and suggest further research on reprogramming the TME, which could offer new strategies to overcome immunotherapy limitations.

Su and Zhang [77] recently published a study based on the TCGA database and genetic alterations of CRGs in colorectal cancer and validated in the GSE41328 dataset, which includes 10 pairs of CRC and adjacent non-tumor tissues [77].

They reported that LIPT1 (Lipoyltransferase 1), PDHA1 (pyruvate dehydrogenase E1 subunit alpha 1), GLS (Glutaminase), and CDKN2A (Cyclin-Dependent Kinase Inhibitor 2A) had significantly higher expression levels in CRC tissues than in normal tissues, while FDX1, DLD (dihydrolipoamide dehydrogenase), and MTF1 (Metal Regulatory Transcription Factor 1) had significantly lower expression levels in CRC tissues than in normal tissues [77]. Moreover, they found that the expression of DLD and CDKN2A were associated with overall survival. They concluded that CRG’s signature could predict outcomes for patients with colorectal cancer [77].

Similar data came from studies by Chen et al. [78]. In total, they collected 698 samples from CRC patients, including 647 tumors and 51 standard samples [78]. Their findings showed that expressions of CDKN2A and DLAT were significantly linked to immune cell infiltration in CRC. They also detected CRG mutations in up to 9.52% of CRC samples [78]. They concluded that cuproptosis was a critical factor in CRC, and targeting the cuproptosis-activating pathway presented a potential individual target for CRC treatment [78].

## 7. Copper and Metastasis of CRC

Colorectal cancer metastasizes upon the detachment of cancer cells from the primary tumor mass, migration, and invasion of the extracellular matrix, intravasation in the bloodstream, and the subsequent extravasation to colonize distant organs.

This progression is facilitated by EMT (epithelial-to-mesenchymal transition, a dynamic and finely regulated biological process in which, following the rewiring of cell gene expression, polarized epithelial cells assume a mesenchymal-like phenotype), angiogenesis, and remodeling of ECM and TME [71]. As reviewed by Focaccio et al. [79], EMT type III occurs during tumor spreading, prompting the acquisition of mesenchymal traits by epithelial cancer cells. Due to this, epithelial cancer cells dissociate from the primary tumor site, invade adjacent tissues, enter the bloodstream, and ultimately establish secondary tumor metastases in distant organs, where the transition from mesenchyme to epithelium (MET) occurs. As a consequence, disseminated cancer cells not only form metastasis, but also favor the acquisition of resistance to treatment, causing poor prognosis. In CRC, the activation of EMT often occurs at the invasive tumor front. Copper and its binding proteins play a vital role in EMT, promoting tumor migration and invasion.

One of the first copper-dependent signaling pathways identified and characterized in the context of cancer is the RAS/RAF/MEK/ERK signaling cascade. In addition, copper is implicated in the modulation of the PI3K/Akt/mTOR signaling cascade, as well as the IL6/JAK/STAT3 axis. In parallel, cuproproteins have been shown to sustain this process by participating in collateral mechanisms.

As reported by Vitaliti et al. [20], the activation of cell signaling pathways through direct interaction with known protein kinases, which exhibit binding domains for copper, supports both tumor growth and dissemination in cancer cells. It is also relevant to the possibility of controlling the cellular levels of copper and its homeostatic regulators.

The activity of lysyl oxidases and lysyl oxidase-like (LOXLs) proteins, a family of enzymes receiving copper from ATP7A, are required for ECM remodeling during EMT. These proteins promote tumor invasiveness by enhancing collagen cross-linking, which in turn triggers integrin clustering in focal adhesions, subsequently activating PI3K signaling and promoting tumor cell invasiveness [80]. LOX increases FAK/SRC phosphorylation and leads to the inhibition of E-calmodulin expression and, thus, making CRC more aggressive and metastatic [81,82,83,84].

Hypoxia is another process triggering alterations that support EMT in cancerous tissues [85]. Hypoxia-inducible factor-1α (HIF-1α), a transcription factor, regulates the transcription of the genes required for fundamental biological processes, including glucose metabolism, cell proliferation, migration, and angiogenesis. HIF-1a (hypoxia-induced factor 1 alpha) also contributes to the metastases through CCS (Copper Chaperone for SOD1), which, with the hypoxia response element (HRE), can result in activating the EMT genes [86]. Furthermore, HIF-1α activates LOX via the PI3K/Akt pathway, creating a synergistic effect that aids in tumor metastasis. Copper activates HIF-1, increasing the transcription of VEGF and ceruloplasmin, which promotes neointima formation [87].

## 8. Therapeutic Potential of Copper in CRC

Given copper’s involvement in the formation and metastasis of colorectal cancer (CRC), targeting copper concentration presents a promising therapeutic approach for CRC. Two potential strategies include the inhibition of copper accumulation using copper chelators and the promotion of cuproptosis through the use of copper ion carriers [88], which are presented in Table 1. Copper chelators such as D-penicillamine and tetrathiomolybdate (TTM), as well as trientine, were studied in CRC and have been reported to inhibit angiogenesis, thereby impairing tumor proliferation and metastasis [89,90,91,92]. In a pilot trial involving 24 patients with metastatic CRC, a combination of tetrathiomolybdate TTM with 5-fluorouracil, leucovorin (IFL), and irinotecan was evaluated [91]. TTM inhibited angiogenesis, and the combination of IFL with TTM was well tolerated, along with the maintenance of the dose intensity of IFL [91]. Trientine, together with methotrexate, significantly reduced the concentration of VEGF and IL-8, and even caused “tumor dormancy”.

Copper ionophores such as disulfiram (DSF), clioquinol (CQ), and bis(thiosemicarbazone) analogs promote cuproptosis by increasing intracellular copper concentration. They facilitate copper removal and induce oxidative stress through natural antioxidant systems like mitochondria, which help to slow the progression of colorectal cancer (CRC) [106]. Additionally, synthetic copper complexes, including proteasome inhibitors, copper pyrithione, and the hinokinin copper complex, enhance the production of reactive oxygen species (ROS) and cause DNA degradation, thereby inhibiting CRC proliferation and metastasis [107,108,109,110]—copper ionophores, as well copper chelators, were investigated in clinical trials, as shown in Table 2.

## 9. Conclusions

Disruptions in copper balance and increased cuproplasia are pivotal in developing colorectal cancer (CRC). The discovery of cuproptosis, a copper-dependent cell death triggered by the lipoylation of mitochondrial enzymes, sheds light on the interaction between copper-induced cellular demise and mitochondrial function [111]. This understanding suggests that targeting copper levels could be an effective strategy for CRC treatment, given its role in cancer formation and spread.

## Figures and Tables

**Table 1:** Copper ionophores and copper chelators as potential therapeutics in various cancer types.

		Cancer Types	Reference
**Cu ionophores**	Diacetyl-bis(N4-methylthiosemicarbazone) (ATSM)	Prostate cancer cells in vitro and in vivo	[93]
	Glyoxal-bis(N4-methylthiosemicarbazone) (GTSM)	Prostate cancer cells in vitro and in vivo	[93,94]
	7-iodo-5-chloro-8-hydroxyquinoline (CQ) Clioquinol	Human breast cancer cells Prostate cancer	[95,96]
	Disulfiram DSF	Drug-resistant prostate cancer	[97]
	Disulfiram DSF	Breast cancer	[98]
	N^′1^,N^′3^-dimethyl-N^′1^,N^′3^-bis(phenylcarbonothioyl) propanedihydrazide—Elesclomol (ES)	Human sarcoma cell lines	[99]
	Elesclomol (ES) nad paclitaxel	Metastatic melanoma	[100]
	Elesclomol (ES) nad paclitaxel	Ovarian, fallopian tube, or primary peritoneal cancer	[101]
	N^′1^,N^′3^-dimethyl-N^′1^,N^′3^-bis(phenylcarbonothioyl) propanedihydrazide elesclomol (ES)	Lung cancer	[102]
**Copper chelators**	Tetrathiomolybdate TM	Human endometrial cancer cell	[103]
	Trientine	Hepatocellular carcinoma	[104]
	D-penicillamine	Melanoma cells	[105]

**Table 2:** Data on the effect of copper ionophores and copper chelation therapy in cancer treatment at the clinical trial level.

NCT Number	Phase	Status	Conditions	Interventions	Enrollment	RESULT
COPPER IONOPHORES						
NCT02963051	I	TERMINATED	Prostate Cancer	Copper Chloride, Disulfiram, Copper Gluconate	9	Study stopped due to lack of efficacy.
NCT03323346	II	RECRUITING	Metastatic Breast Cancer	Disulfiram		
NCT04334837	I	APPROVED FOR MARKETING	Neuroendocrine Tumors	Copper Cu 64 Dotatate		
NCT00742911	I	COMPLETED	Refractory Solid Tumors Involving the Liver	Disulfiram, Copper Gluconate	21	DSF with copper gluconate was well tolerated in patients and not associated with dose-limiting toxicities. No objective responses were observed.
NCT01777919		UNKNOWN	Glioblastoma Multiforme	Temozolomide, Disulfiram, Copper		
NCT04521335	I	TERMINATED	Multiple Myeloma	Disulfiram, Copper Gluconate	2	Closed at PI’s Request
NCT03363659		TERMINATED	Glioblastoma, Glioblastoma Multiforme	Disulfiram, Copper gluconate, Temozolomide	15	
NCT03714555		TERMINATED	Metastatic Pancreatic Cancer	Nab-paclitaxel/gemcitabine Protocol Plus Disulfiram/Copper Gluconate; FOLFIRINOX regimen Plus Disulfiram/Copper Gluconate; Single-agent Gemcitabine regimen Plus Disulfiram/Copper Gluconate	1	Study was closed due to low subject enrollment at site.
NCT05210374		RECRUITING	Relapsed Sarcomas	Disulfiram, Copper Gluconate, Liposomal Doxorubicin (Doxil)		
NCT03034135	II	COMPLETED	Recurrent Glioblastoma	Disulfiram/Copper, Temozolomide (TMZ)	21	Limited activity towards TMZ-resistant IDH-wild-type GBM.
NCT02715609	II	COMPLETED	Glioblastoma Multiforme	Disulfiram, Copper Gluconate, Temozolomide	35	Not known.
NCT03323346	II	RECRUITING	Metastatic Breast Cancer	Disulfiram/Copper		
COPPER CHELATORS						
NCT06134375	I/II	NOT YET RECRUITING	Triple-Negative Breast Cancer|Residual Disease	Tetrathiomolybdate, Capecitabine, Pembrolizumab		
NCT00176774	II	COMPLETED	Colorectal Carcinoma	Irinotecan, 5-Fluorouracil, Leucovorin, Tetrathiomolybdate	24	Reduced angiogenesis in 24 patients.
NCT00003751	II	COMPLETED	Glioblastoma Safety and Superiority of Penicillamine in Radiosensitization of Recurrent Head and Neck Cancer	D-penicillamine	40	Well-tolerated treatment. Reduce copper level did not significantly increase survival in patients with glioblastoma multiforme.
NCT06103617	II	RECRUITING		D-penicillamine		
NCT01178112	I	COMPLETED	Advanced Malignancy	Trientine Carboplatin	56	
NCT02068079	I	WITHDRAWN	BRAF-Mutated Metastatic Melanoma	Trientine with Vemurafenib	0	
NCT03480750	I/II	COMPLETED		Trientine Dihydrochloride, Pegylated Liposomal Doxorubicin, Carboplatin	18	

## Data Availability

No new data were generated.
